# Ruminococcus torques Administration Modestly Alleviates Dietary Selenium Deficiency-Induced Glucose Intolerance in Mature Female Mice

**DOI:** 10.21203/rs.3.rs-7474743/v1

**Published:** 2025-09-29

**Authors:** Ying-Chen Huang, Wen-Hsing Cheng

**Affiliations:** Mississippi State University; Texas Woman’s University

**Keywords:** Selenium deficiency, glucose intolerance, insulin resistance, gut microbiota, Ruminococcus torques

## Abstract

We previously found that dietary selenium (Se) deficiency and age increase the fecal abundance of *Lachnospiraceae* in aged telomere-humanized mice in a sexually dimorphic manner. Although *Lachnospiraceae* are key contributors of short-chain fatty acids to the host, different taxa within this family exert distinct effects on host physiology. Among them, *Ruminococcus torques* has been associated with type 2 diabetes. In the present study, we aimed to determine whether, and how, *R. torques* interacts with dietary Se to influence type 2 diabetes-like symptoms. Sixteen weaning female C57BL/6J mice were fed either a Se-adequate or Se-deficient diet for 26 weeks. From weeks 21 to 25, half of the mice in each dietary group received daily oral gavage of *R. torques* (2 × 10^8^ CFU in 0.2 mL). All mice were euthanized at week 26. Dietary Se deficiency induced glucose intolerance (13%) and insulin resistance (16%) (*P* < 0.05). While *R. torques* administration modestly alleviated glucose intolerance in Se-deficient mice, it did not affect insulin resistance or fasting glucose levels. Se deficiency reduced the relative abundance of *Lactobacillus spp*., *F. prausnitzii*, and *Roseburia spp./E. rectale* in the cecal content, and these taxa were unaffected by *R. torques* treatment. In contrast, Se deficiency increased the relative abundance of *R. torques* and *E. coli* in cecal samples, with *E. coli* levels further elevated by *R. torques* gavage. Notably, *R. torques* oral gavage *1*) decreased SELENOP and GPX1 protein levels in the liver, but not in skeletal muscle, of Se-adequate mice; *2)* increased liver GPX1 protein levels in Se-deficient mice. Altogether, *R. torques* administration modestly alleviates glucose intolerance and increases both liver GPX1 protein levels and cecal *E. coli* abundance in Se-deficient mature female mice with diabetic symptoms.

## Introduction

The *Lachnospiraceae* are a family of anaerobic bacteria within the *Clostridiales* order of the *Firmicutes* phylum, and include species previously classified as part of *Clostridium* cluster XIVa [1, 2]. This family is abundant and dominant in the unperturbed adult gut microbiota [3, 4]. However, only 25% of the genomes are closely related (> 97% similarity) to known species, and 28% do not match any records of existing databases [3]. Interestingly, 19% of sequences are closely related to recently isolated butyrate-producing bacteria from *Clostridium* clusters XIVa and IV, while 18% are related to *Ruminococcus obeum* and *Ruminococcus torques*, which are members of XIVa [3]. *Ruminococcus* is currently considered a polyphyletic genus, with species distributed across two distinct families: the *Ruminococcaceae* and the *Lachnospiraceae* [5]. Based on phenotypic traits, 16S rRNA gene sequence similarity [6], phylogenetic analysis, G + C content of genomic sequence, and DNA-DNA hybridization studies, the species *R. torques*, *R. obeum*, *R. lactaris*, *R. gnavus*, and *R. gauvreauii* are classified within the *Lachnospiraceae* family [7].

Representing a core component of the gut microbiota, *Lachnospiraceae* colonize the intestinal lumen from birth, with their species richness and relative abundance increasing with age. *Lachnospiraceae* have been implicated in obesity and diabetes in both humans and mouse models [8–14]; however, their specific role in type 2 diabetes remains unclear. We have previously shown that long-term dietary selenium (Se) deficiency induces type 2 diabetes-like symptoms in both male and female telomere-humanized mice [15], and increases the fecal abundance of *Lachnospiraceae* at 18 months of age in both sexes, but only in females at 24 months, based on 16 rRNA gene sequence analyses [16]. In contrast, *Akkermansia muciniphila*, a mucin-degrading bacterium that comprises 3–5% of the microbial community in healthy individuals [17], shows the most pronounced age-associated enrichment in response to dietary Se deficiency, but only in male telomere-humanized mice. Within the *Lachnospiraceae* family, *R. torques* is another mucin-degrading bacterium whose abundance is positively associated with irritable bowel syndrome in humans [18, 19]. Because oral gavage of *A. muciniphila* alleviates type 2 diabetes-like symptoms in Se-deficient male mice, and these findings collectively suggest that mucin-degrading bacteria may play a critical role in modulating inflammatory responses at the gut mucosal surface [16]. Therefore, we aimed to investigate the effects of oral administration of *R. torques* on type 2 diabetes-like symptoms induced by dietary Se deficiency, as well as on symbiotic changes involving other mucin-degrading or short-chain fatty acid-producing bacteria in mature female mice. These results may also provide insights into sexually dimorphic responses in Se deficiency-induced type 2 diabetes and gut microbiota alterations.

## Materials and Methods

### Culture of Ruminococcus torques

*R. torques* (ATCC BAA-2281) were cultured under strict anaerobic conditions using the Anoxomat III Jar system (Advanced Instruments, Norwood, MA) in modified reinforced clostridial medium following ATCC protocol. Cultures were washed and concentrated anaerobically in sterile, anaerobic PBS containing 25% (vol/vol) glycerol to a final concentration of 1 × 10^10^ CFU/mL, then immediately frozen and stored at −80°C. Before oral administration, stocks were thawed and diluted anaerobically in sterile PBS with 2.5% glycerol to a final concentration of 1 × 10^9^ CFU/mL.

### Mice, diets, and treatment

**Figure 1:**
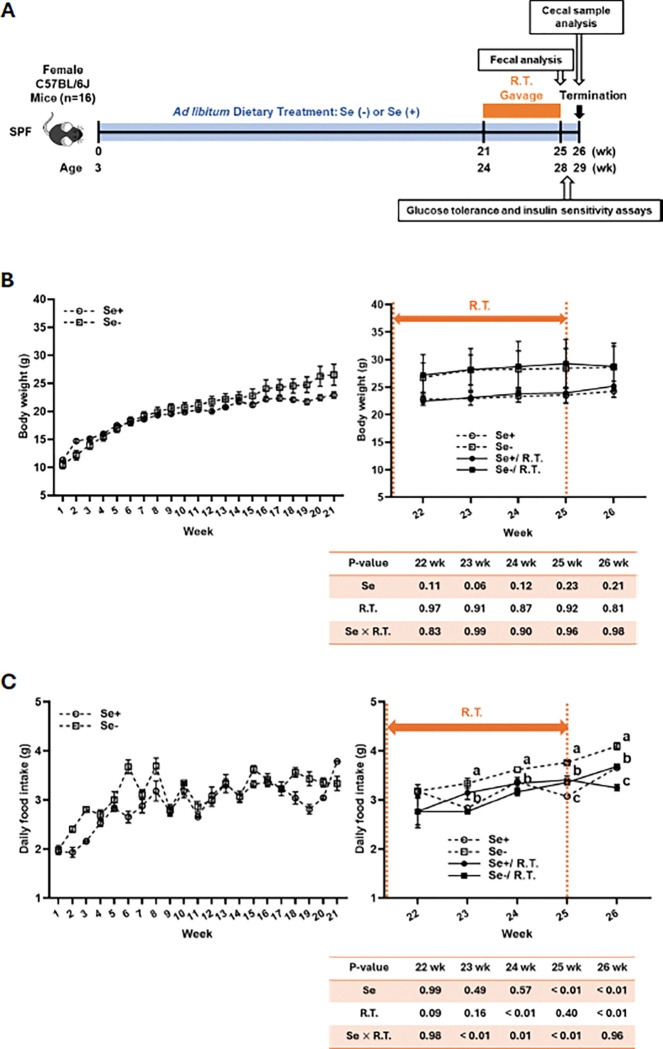
Experimental design, body weight, and food intake in female C57BL/6J mice Schematic diagram illustrating experimental design (A), body weight (B), and food intake (C) in 3-weeks-old female C57BL/6J mice fed either a Se-adequate or Se-deficient diet for 26 weeks. Twenty-one weeks after the start of dietary manipulation, mice received daily oral gavage with *R. torques* or mock treatment for 4 weeks. Values are means ± SEMs (n=4). Means without sharing a common letter at a given time point differ, *P* < 0.05. R.T., *R. torques*; Se+, selenium-adequate diet; Se−, selenium-deficient diet; SPF, specific pathogen-free.

As shown in Fig. 1A, sixteen weaning female C57BL/6J mice were housed under conventional specific pathogen-free (SPF) conditions and fed either a Se-deficient or Se-adequate torula yeast-based purified diet for 26 weeks, as previously described previously [16]. At week 21, mice (n = 4 per group) received a daily oral gavage of either 2 × 10^8^ CFU live *R. torques* or a control vehicle (2.5% glycerol in 200 μL) for 4 weeks. Mice were handled aseptically in a controlled environment with 12-hour light/dark cycle (lights on from 6 p.m. to 6 a.m.), with *ad libitum* access to food and water. Body weight and food intake were monitored weekly. Fresh fecal samples were collected before and after *R. torques* treatment, snap-frozen in liquid nitrogen, and stored at −80°C. Two days after the insulin sensitivity assay, mice were fasted for 6 hours, anesthetized with carbon dioxide, and euthanized by cardiac exsanguination. Liver, skeletal muscle, and cecal contents were collected, rapidly frozen in liquid nitrogen, and stored at −80°C for further analyses. All procedures were approved by the Institutional Animal Care and Use Committee of Mississippi State University.

### Glucose tolerance and insulin sensitivity

At weeks 21 and 26 of dietary intervention, mice fasted for 8 hours were intraperitoneally injected with glucose (1 g/kg body weight) or insulin (0.25 U/kg body weight) (Sigma Aldrich, St. Louis, MO). Blood glucose concentrations were measured using a glucose meter (Bayer Contour Next EZ, Ascensia Diabetes Care US, Inc., Parsippany, NJ) from a drop of tail vein samples collected at 0 (baseline), 0.25, 0.5, 1, 1.5, 2 hours post-injection. Insulin sensitivity was assessed 2 days after glucose tolerance testing. The area under the curve (AUC) was calculated to quantify glucose and insulin responses.

### Bacterial genomic DNA extraction and Quantitative PCR (qPCR) analysis

Bacterial DNA was extracted from cecal contents and feces using the QIAamp PowerFecal Pro DNA Kit (#51804, QIAGEN, Germantown, MD) following the manufacturer’s instructions. Universal primers targeting the V4 region of the bacterial 16S rRNA gene were used for amplification (primer sequences listed in Supplemental Table 1). qPCR was performed in 10 μL reactions using PowerUp^™^ SYBR^™^ Green Master Mix (#A25741, Applied Biosystems, Waltham, MA) on a QuantStudio 5 Real-Time PCR System (#A34322, Applied Biosystems) with the following thermal profile: 95°C for 2 min, followed by 40 cycles of 95°C for 5 sec and 60°C for 30 sec. Total bacterial load in fecal samples was quantified using the ΔCT method. Due to normalization of ΔCT values by subtraction against the control group, two-way ANOVA was not appropriate for analyzing ΔΔCT data, as a designated control could not be applied across two factors. Instead, unpaired t-tests were used for statistical analysis.

### Immunoblotting

Tissues were homogenized in RIPA lysis buffer with protease inhibitors (# sc-24948, Santa Cruz Biotech, Dallas, TX) and centrifuged at 12,000 × g for 10 min at 4°C. Supernatants (30 μg protein per lane) were separated by 14% SDS-PAGE, then transferred to polyvinylidene difluoride membranes. Membranes were incubated overnight at 4°C with primary antibodies (listed in Supplemental Table 2), followed by HRP-conjugated secondary antibodies for 2 hours at room temperature. Signals were developed using Clarity Western ECL substrate, and images were captured and quantified using a Chemidoc-XS system with the volume tool in Image Lab Software (Bio-Rad Lab, Hercules, CA). Protein levels were normalized to albumin, β-tubulin, or total AKT.

### Statistical analysis

Data are presented as means ± SEM. Most datasets were analyzed by two-way ANOVA followed by Tukey’s post hoc test, except for qPCR-based assays, which were evaluated using unpaired *t*-tests. Statistical analyses were performed using SAS (version 9.4) and GraphPad Prism (version 8.0). A significance level of α = 0.05 was used for all tests.

## Results

### Body weight and food intake

Weaning female C57BL/6J mice exhibited steady increases in body weight (103–153%; *P* < 0.05; Fig. 1B) and food intake (56–70%; *P* < 0.05; Fig. 1C) over the 21-week dietary intervention. In mice receiving control oral gavage, dietary Se deficiency increased food intake (8–18%; *P* < 0.05) but had no effect on body weight. Daily oral gavage with *R. torques* resulted in a deduction in food intake in Se-deficient mice at weeks 23–26 (10–17%; *P* < 0.05), without affecting body weight. In *R. torques*-gavaged mice, dietary Se deficiency increased food intake at week 26 (13%; *P* < 0.05) but did not alter body weight.

### Changes in the abundance of specific genera by dietary Se deficiency and R. torques oral gavage in mice

**Figure 2:**
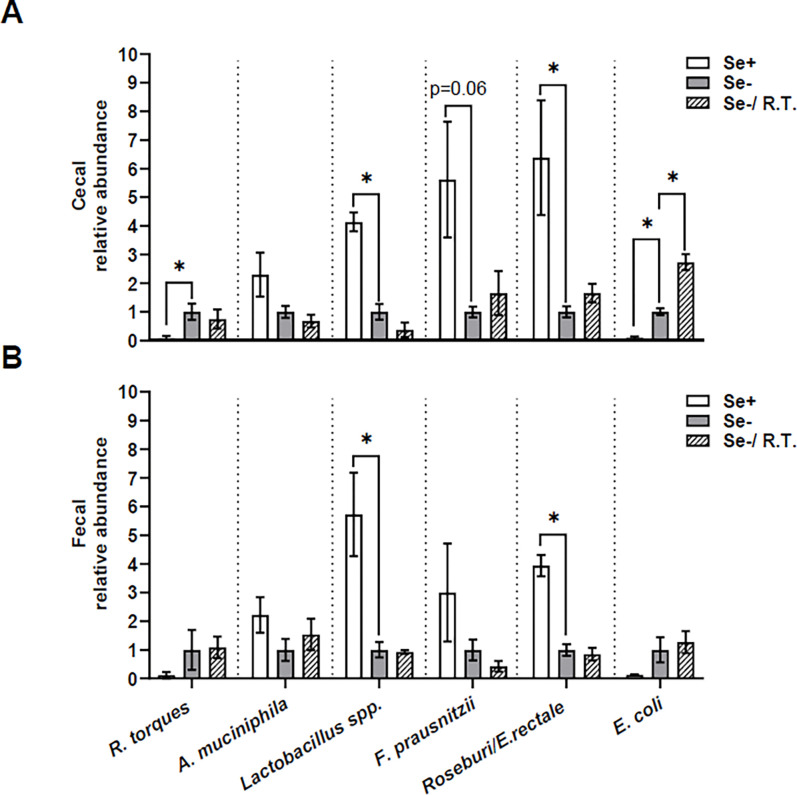
Fecal and cecal abundance of six bacteria taxa Relative abundance of *R. torques, A. muciniphila, Lactobacillus spp., F. prausnitzii, Roseburia/E. rectale,* and *E. coli* in cecal (A) and fecal (B) samples from female C57BL/6J mice fed either a Se-adequate or Se-deficient diet. *R. torques* oral gavage was administered to Se-deficient mice (See Figure 1A for detailed experimental design). Values are means ± SEMs (n = 4). *P < 0.05. R.T., *R. torques*; Se+, selenium-adequate diet; Se−, selenium-deficient diet.

In Se-adequate mice, oral gavage with *R. torques* was associated with apparent increases (3- to 5-fold) in the abundances of *R. torques*, alongside decreases (52–82%; *P* < 0.05) in *Lactobacillus spp*. and *Roseburia/E. rectale* in the cecal and fecal contents (Supplemental Fig. 1). In mice receiving control oral gavage, dietary Se deficiency increased (*P* ≤ 0.06) the abundances of *R. torques* (10.9-fold) and *E. coli* (9.5-fold), while decreasing that of *Lactobacillus spp.* (3.1-fold), *F. prausnitzii* (4.6-fold), and *Roseburia/E. rectale* (5.3-fold) in cecal content (Fig. 2A). A similar trend was observed in fecal content (Fig. 2B), but only *Lactobacillus spp*. (4.7-fold) and *Roseburia/E. rectale* (2.9-fold) reached statistical significance (*P* < 0.05). Oral gavage with *R. torques* had no effect on these Se deficiency-induced alterations, except for a 1.7-fold increase (*P* < 0.05) in cecal *E. coli* abundance. In mice receiving oral gavage with *R. torques*, dietary Se deficiency increased the relative abundance of *E. coli* in both cecal and fecal contents (4- to10-fold; *P* < 0.05) and decreased that of fecal *Roseburia/E. rectale* by 54% (*P* < 0.05), but not in other taxa (supplemental Fig. 2).

### Modest alleviation by R. torques of dietary Se deficiency-induced glucose intolerance, but not insulin insensitivity, in conventional mice

**Figure 3:**
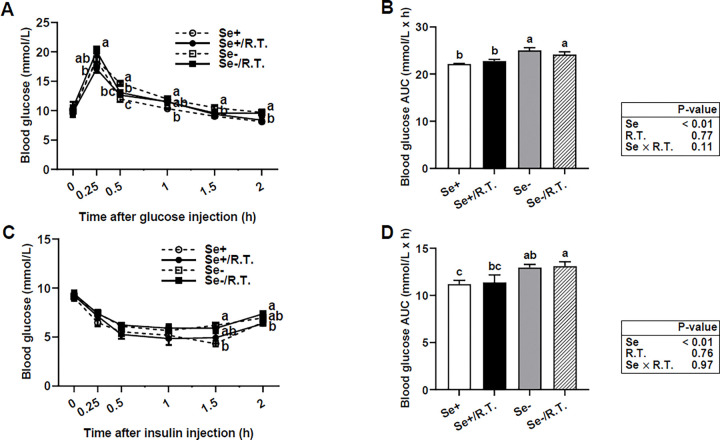
Glucose tolerance and insulin sensitivity assays in female C57BL/6J mice Blood glucose levels were measured following intraperitoneal injection of glucose (1 g/kg; A, B) or insulin (0.25 U/kg; C, D) in female C57BL/6J mice fed either a Se-adequate or Se-deficient diet and given *R. torques* or mock oral gavage (see Figure 1A for detailed experimental design). Mice were fasted 8 hours prior to glucose or insulin injection. The average area under the curve was calculated from the data in panels A and C, with units expressed as mmol • h • L−1 (B and D). Values are means ± SEMs (n=4). Means without sharing a common letter at a given time point differ, *P* < 0.05. AUC, average area under the curve; R.T., *R. torques*; Se+, selenium-adequate diet; Se−, selenium-deficient diet.

Se-deficient female mice at ~ 7 months of age exhibited glucose intolerance (Fig. 3A and B) and insulin resistance (Fig. 3C and D) over a 2-hour period following injections. However, fasting blood glucose concentrations were not affected by dietary Se deficiency or by *R. torques* oral gavage (Fig. 3A and C). While oral gavage with *R. torques* modestly alleviated (11% improvement; *P* < 0.05) Se deficiency-induced glucose intolerance at 0.5 h post-injection (Fig. 3A), it had no significant effect on overall glucose tolerance or insulin sensitivity across the entire time course in either the Se-deficient or Se-adequate mice.

### R. torques oral gavage and dietary Se deficiency differentially affect selenoprotein expression in muscle and liver

We next assessed body Se status in the liver and skeletal muscle by evaluating protein expression levels of selected selenoproteins. Western blot analysis revealed that dietary Se deficiency reduced (*P* < 0.05) the protein levels of GPX1 by 41% and SELENOW by 89% in muscle; whereas SELENOH and SELENOP expression remained unaffected (Fig. 5A). In the liver (Fig. 5B), Se deficiency decreased (*P* < 0.05) protein levels of SELENOP by 50%, GPX1 by 86%, SELENOH by 56%, and SELENOW by 88%. Notably, the reduction in hepatic GPX1 was partially reversed by *R. torques* oral gavage (1.6-fold increase; *P* < 0.05). In Se-adequate mice, oral gavage of *R. torques* reduced (*P* < 0.05) hepatic levels of both SELENOP and GPX1 by 27%, but had no effect on SELENOH or SELENOW in the liver, nor on any selenoprotein expression in muscle.

## Discussion

We have previously shown that long-term dietary Se deficiency induces type 2 diabetes-like symptoms in telomere-humanized mice aged 12 and 18 months in both sexes [15], as well as middle-aged and mature wild-type male mice [16, 20]. Here, compared to wild-type male mice aged 4–7 months [16], dietary Se deficiency in age-matched females induces glucose intolerance and insulin resistance to a lesser extent. Consistent with our findings, there is evidence that females are generally more insulin-sensitive than males [21, 22], and women have decreased susceptibility to fatty acid–induced peripheral insulin resistance [23]. In rodent models, males typically exhibit more pronounced diabetes symptoms than females [24–27]. These sex-related differences in type 2 diabetes-like symptoms may be partially attributed to the actions of estrogen and testosterone. For example, decreases in estrogen and increases in testosterone levels during menopause are associated with a loss of subcutaneous fat, a gain of visceral fat, and increased insulin resistance [28]. Indeed, we have previously shown that Se status under Se deficiency is tissue-specific and dependent on sex and age [29].

In contrast to *A. muciniphila* [16], oral gavage with *R. torques* only modestly alleviates Se deficiency-induced type 2 diabetes symptoms. An intriguing question arises: why do these two mucin-degrading bacteria have opposing impacts on Se deficiency-induced metabolic symptoms? Although some mucin-degrading bacteria, such as *A. muciniphila*, are associated with health benefits, their close proximity to the intestinal epithelium may pose risks to host cells by compromising the gut barrier. It has been suggested that a reduction in cecal *R. torques* abundance in response to *Lactobacillus acidophilus* treatment may improve reproductive performance of broiler chickens by reducing the incidence of pasty vent, a stress-induced condition characterized by dry, cake-like droppings around the vent of baby chicks [30]. Supporting this observation, mucin degradation has been proposed to be pathogenic, as the loss of the protective mucus layer may increase the exposure of gastrointestinal epithelial cells to pathogens [31]. Moreover, the abundance of total mucosa-associated bacterial 16S rRNA genes is elevated in inflammatory bowel disorder, suggesting an increased availability of digestible endogenous mucus that may, in turn, sustain the growth of non-mucolytic mucosa-associated bacteria [32]. These findings highlight the need for further investigation into the role of mucin in shaping microbial community dynamics and host–microbe interactions.

*R. torques* has been reported to be correlated with markers of insulin resistance in humans [33]. In our study, *R. torques* abundance was increased in mice fed a Se-deficient diet compared to those on a Se-adequate diet. However, our results indicate that oral administration of *R. torques* modestly improves glucose intolerance but has no effect on insulin resistance in Se-deficiency female mice. We speculate that *1)* female mice may exhibit greater baseline insulin sensitivity, potentially masking any mild effects of *R. torques*; *2)* interactions between *R. torques* and host selenoproteins may counterbalance each other’s influence on type-2 diabetes pathogenesis; 3) the effects of *R. torques* on Se deficiency-induced type 2 diabetes may be age-dependent, becoming apparent only in middle-aged or older mice, but not in younger mice.

Although a recent study using the type strain *R. torques* ATCC 27756 (BSL-1) demonstrated a pronounced alleviation of glucose intolerance in obese mice via oral gavage [2], we employed a non-type strain, *R. torques* ATCC BAA-2281 (BSL-2), and observed only a modest improvement in Se deficiency-induced glucose intolerance. In addition to the fact that ATCC BAA-2281 has been minimally characterized, we selected this strain to test its effect on type 2 diabetes-like symptoms because it is deposited in ATCC as a *Lachnospiraceae* bacterium. We have previously shown that dietary Se deficiency enriches the relative abundance of *Lachnospiraceae* in aged telomere-humanized mice in a sexually dimorphic manner [16]. To better understand the differing efficacy of these two *R. torques* strains in modulating glucose tolerance, future studies should consider factors such as host obesity status, strain-level genetic variation, and the underlying etiology of type 2 diabetes.

Other gut bacteria may respond to *R. torques* oral gavage and influence outcomes related to glucose intolerance and insulin resistance in Se-deficient mice. Indeed, Se deficiency in females decreases the abundance of *Lactobacillus spp.*, *F. prausnitzii*, and *Roseburia spp./E. rectale*, while increasing the abundance of *R. torques* and *E. coli* in the cecal content. However, *R. torques* oral gavage results in increased *E. coli* abundance in Se-deficient mice and decreased *Lactobacillus spp*. and *Roseburia spp./E. rectal* abundance in Se-adequate mice. These findings are in contrast to those observed with *A. muciniphila* oral gavage, suggesting that these two mucin-degrading bacteria impact gut homeostasis and host health through distinct mechanisms.

Altogether, our results show that the administration of the *R. torques* ATCC BAA-2281 strain only modestly alleviates glucose intolerance in Se-deficient female mice, and that changes in five other bacterial taxa display patterns distinct from those observed with *A. muciniphila* oral gavage in male mice [16]. Further studies are needed to deepen our understanding and provide mechanistic insight into how *R. torques* influences Se status and type 2 diabetes in the host, as well as how Se may prevent the early onset of type 2 diabetes in a sexually dimorphic manner.

## Supplementary Material

Supplementary Files

This is a list of supplementary files associated with this preprint. Click to download.

• SupplementalFigures12final.pdf

• SupplementalTablesfinal.pdf

## Figures and Tables

**Figure 4:**
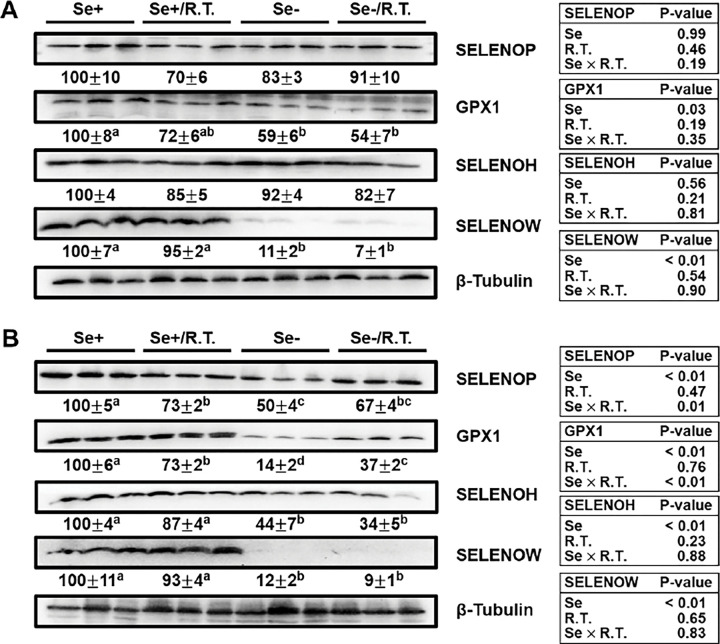
Western blot analysis of body Se status in female C57BL/6J mice Selenoprotein expression in skeletal muscle (A) and liver (B) of female C57BL/6J mice fed either a Se-adequate or Se-deficient diet and given *R. torques* or mock oral gavage (see Figure 1A for detailed experimental design). Protein levels were normalized to β-tubulin and expressed as a percentage of the Se-adequate control group. Values are means ± SEMs (n=4). Means without sharing a common letter differ, *P* < 0.05. GPX1, glutathione peroxidase 1; R.T., *R. torques*; Se+, Se-adequate diet; Se−, Se-deficient diet; SELENOH, selenoprotein H; SELENOP, selenoprotein P; SELENOW, selenoprotein W.

## Abbreviations

AKT: mouse thymoma viral protooncogene
GPX1: glutathione peroxidase 1
GPX3: glutathione peroxidase 1
Se: selenium
SELENOH: selenoprotein H
SELENOP: selenoprotein P
SELENOW: selenoprotein W
SCFA: short-chain fatty acid
